# Probing conformational states of glutaryl-CoA dehydrogenase by fragment screening

**DOI:** 10.1107/S1744309111014436

**Published:** 2011-08-13

**Authors:** Darren W. Begley, Douglas R. Davies, Robert C. Hartley, Stephen N. Hewitt, Amanda L. Rychel, Peter J. Myler, Wesley C. Van Voorhis, Bart L. Staker, Lance J. Stewart

**Affiliations:** a Seattle Structural Genomics Center for Infectious Disease (http://www.ssgcid.org), USA; b Emerald BioStructures Inc., 7869 NE Day Road West, Bainbridge Island, WA 98110, USA; c Department of Allergy and Infectious Diseases, School of Medicine, University of Washington, Box 356423, Seattle, WA 98195, USA; d Department of Biology, University of Washington, Seattle, WA 98195-1800, USA; e Seattle Biomedical Research Institute, 307 Westlake Avenue North, Suite 500, Seattle, WA 98109, USA; f Departments of Global Health, Medical Education and Biomedical Informatics, School of Medicine, University of Washington, Box 357230, Seattle, WA 98195, USA; g Departments of Microbiology, Global Health, and Allergy and Infectious Diseases, School of Medicine, University of Washington, Seattle, WA 98195, USA

**Keywords:** *Burkholderia pseudomallei*, glutaryl-CoA dehydrogenase, glutaric acidemia, fragment screening, flavoproteins, pantothenate, glutaryl-CoA, crotonyl-CoA, flavin adenine dinucleotide, SSGCID

## Abstract

Glutaric acidemia type 1 is an inherited metabolic disorder which can cause macrocephaly, muscular rigidity, spastic paralysis and other progressive movement disorders in humans. The defects in glutaryl-CoA dehydrogenase (GCDH) associated with this disease are thought to increase holoenzyme instability and reduce cofactor binding. Here, the first structural analysis of a GCDH enzyme in the absence of the cofactor flavin adenine dinucleotide (FAD) is reported. The apo structure of GCDH from *Burkholderia pseudomallei* reveals a loss of secondary structure and increased disorder in the FAD-binding pocket relative to the ternary complex of the highly homologous human GCDH. After conducting a fragment-based screen, four small molecules were identified which bind to GCDH from *B. pseudomallei*. Complex structures were determined for these fragments, which cause backbone and side-chain perturbations to key active-site residues. Structural insights from this investigation highlight differences from apo GCDH and the utility of small-molecular fragments as chemical probes for capturing alternative conformational states of preformed protein crystals.

## Abstract 2

The first crystal structure is reported of a glutaryl-CoA dehydrogenase in the apo state without flavin adenine dinucleotide cofactor bound. Additional structures with small molecules complexed in the catalytic active site were obtained by fragment-based screening.

## 1. Introduction

Glutaryl-CoA dehydrogenase (GCDH) is an acyl-CoA dehydro­genase (ACDH) which catalyzes an intermediate step in the metabolic breakdown of lysine and tryptophan (Gomes *et al.*, 1981 ▶; Kim & Miura, 2004 ▶; Lenich & Goodman, 1986 ▶; Goodman *et al.*, 1995 ▶). Unlike other flavoproteins within this family, GCDH (EC 1.3.99.7) performs α,β-dehydrogenation as well as decarboxylation of its sub­strate glutaryl-CoA to yield the product crotonyl-CoA (Dwyer *et al.*, 2000 ▶). Transfer of electrons from the glutaryl-CoA precursor to the electron-transfer flavoprotein is mediated by the cofactor flavin adenine dinucleotide (FAD) in both prokaryotic and eukaryotic electron-transport chains (Tsai & Saier, 1995 ▶). Defects in GCDH are reponsible for glutaric acidemia type 1 (GA1), an inherited metabolic disorder which prevents the complete breakdown of lysine and tryptophan (Strauss *et al.*, 2003 ▶; Strauss & Morton, 2003 ▶; Goodman *et al.*, 1998 ▶). This disease manifests as macrocephaly and bleeding abnormalities in newborns, as well as muscular rigidity, spastic paralysis and other progressive movement disorders in older individuals (Strauss *et al.*, 2003 ▶; Hedlund *et al.*, 2006 ▶). Treatments for this disease include intravenous delivery of carnitine, which is depleted as a secondary effect of GA1, and a restricted-protein diet to limit lysine and tryptophan buildup in the circulatory system. The statistical chance of inheriting GA1 worldwide is 1:100 000, but is as high as 1:300 in Old Order Amish communities in Pennsylvania and other ethnic societies (Morton *et al.*, 1991 ▶). To date, over 80 mutations, including missense, nonsense and intronic variations, have been associated with GA1 in patients (Keyser *et al.*, 2008 ▶; Westover *et al.*, 2003 ▶; Goodman *et al.*, 1998 ▶). However, controversy persists con­cerning attempts to connect phenotype to specific genotypic markers of the disease, as patients symptomatic for GA1 sometimes do not present with GCDH deficiency (Westover *et al.*, 2003 ▶; Christensen *et al.*, 2004 ▶; Garcia *et al.*, 2008 ▶).

**Figure 1:**
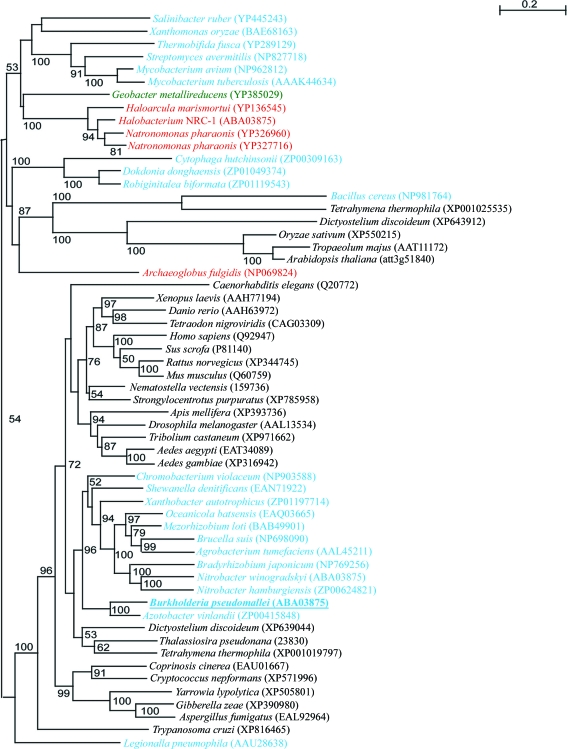
A bootstrapped nearest-neighbour-joining phylogenetic tree of glutaryl-CoA dehydrogenases from a selection of eukaryotic (black), archaeal (red) and aerobic (cyan) bacterial species, plus one prokaryotic obligate anaerobe (green). A subtree for *B. pseudomallei* (underlined) and other related prokaryotes is well supported within eukaryota and is suggestive of lateral gene transfer. The alignment was performed using *ClustalX*2 v.2.0 (Chenna *et al.*, 2003 ▶).

Sequence differences between mammalian and bacterial GCDHs can be large for specific anaerobes, in which GCDH plays a crucial role in benzoyl-CoA degradation. Preliminary evidence suggests that certain obligate anaerobes possess two distinct enzymes which separately perform the dehydrogenation and decarboxylation steps (Wischgoll *et al.*, 2009 ▶, 2010 ▶). However, a detailed phylogenetic analysis of the ACDH superfamily shows certain bacterial GCDH genes nesting closer to eukaryotic sequences than to genes from other bacteria, suggesting a lateral gene transfer from eukaryotes to proteobacteria (Swigonová *et al.*, 2009 ▶). We performed a similar analysis to include the protein sequence of GCDH from *Burkholderia pseudomallei* and found that this enzyme was situated within the eukaryotic gene-transfer group (Fig. 1 ▶). Together, the sequence correlation and high structural homology (see below) suggest that structural insights gained for GCDH from *B. pseudomallei* will have consequences related to the human enzyme.

At the time of writing, seven structures of GCDH are available in the Protein Data Bank (PDB) from the human, *Thermus thermophilus* and *Desulfococcus multivorans* gene products, all of which contain the bound cofactor FAD (Fu *et al.*, 2004 ▶; Rao *et al.*, 2007 ▶; Thorpe & Kim, 1995 ▶; Wischgoll *et al.*, 2010 ▶). Here, we report the first structures of GCDH from *B. pseudomallei* (BpGCDH), including the first apo structure of any GCDH characterized in the absence of FAD. The results of this investigation allow a detailed comparison between human and bacterial GCDH and determination of the structural consequences associated with the apo structure. Among these differences, a tyrosine side chain adjacent to the catalytic glutamate residue in apo BpGCDH is rotated nearly 180° from its position in the human ternary complex and appears to block the flavin-binding site. By soaking our Fragments of Life small-molecule library (Davies *et al.*, 2009 ▶; Begley *et al.*, 2011 ▶) into crystals of apo BpGCDH, complexes were formed whose crystal structures showed backbone hydrogen-bond interactions replaced by the catalytic glutamate side chain. These structures shed light on the structural flexibility available to GCDH and demonstrated the use of small-molecular fragments as chemical probes for capturing alternative conformational states.

## 2. Experimental methods

### 2.1. Protein expression and purification

Full-length glutaryl-CoA dehydrogenase (GCDH; UniProt Q3JP94) was obtained from purified *B. pseudomallei* strain 1710b genomic DNA, which was kindly provided by Rajinder Kaul from the University of Washington. The gene was amplified using the following primer sequences: FWD primer 5′-GGGTCCTGGTTCGATGGCT­GCCGCAACCTTCCAC-3′ and REV primer 5′-CTTGTTCGTGC­TGTTTATTAGAAGAACGCCTGAATCCCCGTC-3′ (Invitrogen). The PCR conditions were 35.2 µl deionized H_2_O (Sigma, catalog No. W3513), 5 µl Expand High Fidelity Buffer (10×) with 15 m*M* MgCl_2_ (Roche), 0.4 µl 25 m*M* dNTPs (Qiagen, catalog No. 201912), 0.4 µl Expand High Fidelity Enzyme Mix (Roche, catalog No. 11732641001), 4 µl FWD primer, 4 µl REV primer and 1 µl (10 ng) *B. pseudomallei* 1710b genomic DNA. Thermal cycling conditions were 367 K for 2 min followed by 30 cycles of 367 K (30 s), 333 K (1 min) and 345 K (4 min 30 s) and a final extension of 345 K for 10 min using a Bio-Rad MJ Research PTC-200 thermal cycler. PCR amplicons were run on agarose gel to verify the expected size of the amplified gene, which was followed by excision and extraction from the gel using a QiaQuick kit (Qiagen, catalog No. 28181).

The purified PCR product was cloned into the expression vector AVA0421 with a cleavable 6×His fusion tag followed by the human rhinovirus 3C protease-cleavage sequence (MAHHHHHHMGTL­EAQTQGPGS-ORF for the entire tag sequence N-terminal to the target gene) by ligation-independent cloning (LIC; Aslanidis & de Jong, 1990 ▶). Briefly, purified PCR product was treated with T4 polymerase in the presence of the single nucleotide dATP, creating overhangs, and then annealed with compatible, linearized and T4-treated AVA0421 vector (Mehlin *et al.*, 2006 ▶). Annealed vector and insert were transformed into NovaBlue competent cells (Novagen, catalog No. 71011-4) and plated on LB agar (BD Difco LB Agar Miller-BD, catalog No. 244520) with 50 µg ml^−1^ each of ampicillin (Anatrace, catalog No. A1000) and carbenicillin (Duchefa Bio­chemie, catalog No. C0109.0025) to select for cells carrying the expression plasmid. The presence of the insert was verified by colony PCR under the above conditions, but the colony itself was resuspended in water and used as template instead of purified DNA. Plasmid DNA was purified (QIAprep Turbo Miniprep kit; Qiagen, catalog No. 27191) from 1 ml overnight cultures and then transformed into the expression host *Escherichia coli* BL21 Star (DE3) (Invitrogen, catalog No. C6010-03).

2 l cultures of GCDH were grown in a LEX bioreactor (Harbinger) at 293 K in auto-induction medium (Studier, 2005 ▶). After 72 h of growth, the culture was pelleted in a Sorvall RC 12BP fitted with an H-12000 rotor by spinning for 20 min at 4300 rev min^−1^, after which the cell paste was harvested and flash-frozen in liquid nitrogen. Unless otherwise noted, the protein was purified at room temperature. To prepare protein samples, the cell paste was solubilized on ice in lysis buffer (25 m*M* HEPES, 500 m*M* NaCl, 5% glycerol, 30 m*M* imidazole, 0.5% CHAPS, 10 m*M* MgCl_2_, 1 m*M* TCEP, 250 ng ml^−1^ AEBSF pH 7.0) with 0.01 g lysozyme. The cell paste was sonicated on ice for 30 min (100 W; cycles of 15 s pulse-on and 15 s pulse-off; Virtis, catalog No. 408912). After sonication, samples were treated with Benzonase (500 U total) and then centrifuged for 1 h at 277 K (14 000 rev min^−1^ in a Sorvall SLA-1500 rotor) to clarify cell debris. The protein was purified by immobilized metal ion-affinity chromatography on pre-equilibrated (25 m*M* HEPES pH 7.0, 500 m*M* NaCl, 5% glycerol, 30 m*M* imidazole, 1 m*M* TCEP and 0.025% azide) 5 ml Ni Sepharose columns (HisTrap FF; GE Healthcare, catalog No. 17-­5255-01) using an ÄTKAexplorer. After thorough washing, bound protein was eluted from the nickel column by the addition of elution buffer (25 m*M* HEPES pH 7.0, 500 m*M* NaCl, 5% glycerol, 1 m*M* TCEP, 250 m*M* imidazole and 0.025% azide). Fractions from nickel-affinity chromatography were analyzed for protein content by SDS–PAGE and pooled. His-MBP-3C protease was added to the target protein at a 1:50(*w*:*w*) ratio in order to remove the N-terminal 6×His tag. The protease was incubated overnight at 277 K in 3C buffer (20 m*M* HEPES, 200 m*M* NaCl, 5% glycerol, 1 m*M* TCEP pH 7.5) and the cleaved protein was passed over Ni resin beads (Ni Sepharose 6 Fast Flow; GE Healthcare, catalog No. 17-5318-02) to remove noncleaved protein, the cleaved 6×His tag, His-tagged 3C protease and any contaminants which bind to nickel. After cleavage, the construct generated retains a GPGS- sequence, followed by the first N-terminal methionine of GCDH. Clarified cleaved protein was then further purified by size-exclusion chromatography (SEC; HiLoad 26/60 Superdex 75; GE Healthcare, catalog No. 17-1071-01) using an ÄTKAprime in SEC buffer (25 m*M* Tris pH 8.0, 200 m*M* NaCl, 1% glycerol and 1 m*M* TCEP). SEC fractions were analyzed by SDS–PAGE, showing a single band at the expected elution volume. The highest intensity SEC fractions were pooled and concentrated to approximately 26 mg ml^−1^ using Amicon Ultra-15 concentrators with a molecular-weight cutoff of 3000 Da (Fisher, catalog No. UFC901096). Protein samples were aliquoted in 100 µl volumes, flash-frozen in liquid nitrogen and stored at 193 K.

### 2.2. Crystallization and fragment screening

Sitting-drop vapor-diffusion crystallization trials were set up at 289 K using the JCSG+, PACT and Cryo Full sparse-matrix screens from Emerald BioSystems and the Crystal Screen HT sparse-matrix screen from Hampton Research. Protein solution (0.4 µl) was mixed with reservoir solution (0.4 µl) and equilibrated against 100 µl reservoir solution using 96-well Compact Jr plates from Emerald BioSystems. Crystals grew in several conditions, but those used in X-­ray data collection and structure determination were obtained using a reservoir solution consisting of 20% PEG 3000, 100 m*M* HEPES, 200 m*M* NaCl pH 7.5. Crystals of apo BpGCDH were cryoprotected using 25%(*v*/*v*) ethylene glycol in the reservoir solution prior to freezing in liquid nitrogen and data collection.

Fragment screening was performed on preformed GCDH crystals with a metabolome-derived small-molecule library of 1440 fragments following previously published methods (Davies *et al.*, 2009 ▶; Begley *et al.*, 2011 ▶). Briefly, pools of up to eight fragment ligands were prepared from concentrated methanol stock solutions with each ligand at 6.25 m*M* in the mixture. One 1.0 µl drop of each methanol pool was then added to an empty sitting-drop well and the methanol was allowed to completely evaporate, leaving behind a dry film of fragment compounds. The fragments were resuspended in 1.0 µl reservoir solution and 2–3 crystals of apo BpGCDH were transferred to each well, with 20 µl reservoir solution being added to each reservoir prior to sealing. After 7–14 d of soaking at 289 K, fragment-soaked crystals were dipped into cryoprotectant solution prior to freezing in liquid nitrogen and data collection. The cryoprotectant consisted of 0.25 µl ethylene glycol and 0.75 µl reservoir solution, with a fresh preparation of 6.25 m*M* resuspended fragments present. All fragments found to bind crystals of BpGCDH in mixtures were followed up by individual fragment soaks at 10–25 m*M* followed by cryoprotection and X-ray data collection to confirm the identity of each small molecule.

### 2.3. Data collection and structure determination

Preliminary data sets were collected in-house using a Rigaku SuperBright FR-E+ X-ray generator with Osmic VariMax HF optics and a Saturn 944+ CCD detector. The final data sets for the individual BpGCDH structures discussed in this work were collected on the APS 21-ID-D, APS 23-ID-D and ALS 5.0.3 synchotron beamlines and were reduced with *HKL*-2000 (Otwinowski & Minor, 1997 ▶). The apo structure was solved by molecular replacement using *Phaser* from the *CCP*4 suite of programs (Winn *et al.*, 2011 ▶) with molecule *A* from the structure of human GCDH (PDB entry 1siq; Fu *et al.*, 2004 ▶) as the search model. All crystal structures were initially rebuilt with *ARP*/*wARP* (Langer *et al.*, 2008 ▶), followed by numerous reiterative rounds of refinement in *REFMAC* (Murshudov *et al.*, 2011 ▶) and manual model building using the *Crystallographic Object-Oriented Toolkit* (*Coot*; Emsley & Cowtan, 2004 ▶). For direct fragment-mixture screening by crystallography, unmodeled electron density with (*F*_o_ − *F*_c_) > 2.5σ and (2*F*_o_ − *F*_c_) > 1.0σ was first verified against the model protein structure using *Coot*. Unmodeled electron density that was likely to be protein or buffer components was ignored and the remaining density regions were examined for shape complementarity with individual fragments in a given mixture. Individual fragment-soaking experiments were then conducted and new data sets were collected to confirm the identity of each bound small-molecule fragment. Fragment-bound structures were solved by molecular replacement from the apo BpGCDH structure, using *CCP*4 to generate small-molecule structures. Each structure was evaluated using *MolProbity* (Chen *et al.*, 2010 ▶) and manually checked by internal peer review prior to structure validation and deposition in the Protein Data Bank (Berman *et al.*, 2000 ▶, 2003 ▶). Data-collection (Table 1 ▶) and refinement (Table 2 ▶) statistics are listed for apo BpGCDH and four fragment-bound complexes.

## 3. Results and discussion

### 3.1. The apo structure of GCDH from *B. pseudomallei*

**Figure 2:**
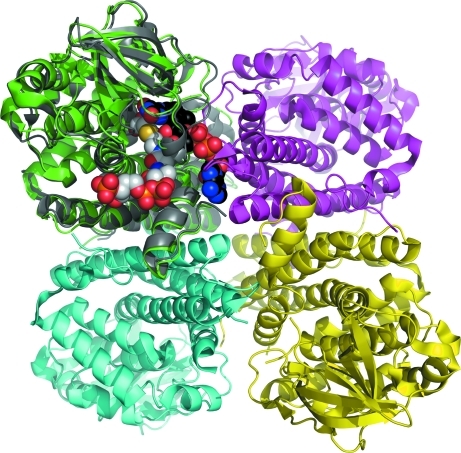
Tetrameric glutaryl-CoA dehydrogenase from *B. pseudomallei* (BpGCDH; PDB entry 3eom) overlaid with one protomer of human glutaryl-CoA dehydrogenase (PDB entry 1sir; Fu *et al.*, 2004 ▶). The BpGCDH tetramer (green, cyan, magenta and yellow) is in the apo state, while the hGCDH protomer (gray) is in a ternary complex with the cofactor FAD (black) and the substrate mimic *S*-4-nitrobutyryl-CoA (NBC; white).

**Figure 3:**
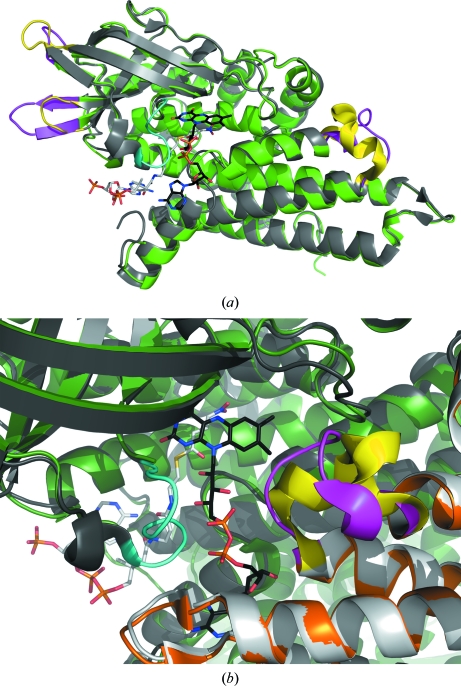
(*a*) A single protomer of apo BpGCDH (green; PDB entry 3eom) overlaid with the human ternary complex (gray; PDB entry 1sir; Fu *et al.*, 2004 ▶) containing FAD (black) and the substrate mimic NBC (white). Regions of structural difference between the two protomers (bacterial, magenta; human, yellow and cyan) are largely contained within terminal loop regions. (*b*) The interface between two protomers of bacterial (green/orange) and human (dark gray/white) GCDH. The human ternary complex reveals an α-helical contact region between protomers (yellow) which is a less ordered loop in BpGCDH (magenta). Density modeled on the opposite protomer in the human complex (cyan) is too disordered to model in the bacterial apo crystal data set.

Like other ACDHs, GCDH from *B. pseudomallei* (BpGCDH) exists as a homotetramer with tetrahedral symmetry and possesses an overall fold similar to that of the human enzyme (Fu *et al.*, 2004 ▶). Each protomer of BpGCDH consists of an N-terminal solvent-facing region of five α-helices followed by a flattened seven-stranded pseudo-β-barrel and a C-terminal α-helix core (Fig. 2 ▶). The C-terminal domain comprises much of the tetrameric binding surface and forms parts of both the FAD and the glutaryl-CoA binding pockets. Residues that are critical for enzymatic activity in the human enzyme are conserved in the bacterial sequence, including the catalytic glutamate Glu374 (Glu370 in human) which abstracts an α proton from the substrate (Fu *et al.*, 2004 ▶; Rao *et al.*, 2007 ▶). At the time of writing, all GCDH structures in the PDB possess FAD bound in the cofactor-binding pocket (PDB entries 1siq, 1sir, 2r0m, 2r0n, 2eba, 3mpi and 3mpj; Fu *et al.*, 2004 ▶; Rao *et al.*, 2007 ▶; T. S. Kumarevel, P. Karthe, S. Kuramitsu & S. Yokoyama, unpublished work; Wischgoll *et al.*, 2010 ▶). None of these structures report FAD as a crystallization additive, despite its presence in the final structure. This suggests that the cofactor is usually carried through purification, with sufficient binding affinity for GCDH to remain bound through multiple purification steps (Fu *et al.*, 2004 ▶; Rao *et al.*, 2007 ▶; Thorpe & Kim, 1995 ▶; Wischgoll *et al.*, 2010 ▶). In our study, overexpression of BpGCDH in *E. coli* and standard protein-purification methods did not yield crystals with FAD bound (see *Methods* [Sec sec2]). The absence of electron density in the cofactor-binding pocket of multiple BpGCDH data sets, together with key differences from the human ternary complex, confirm this result. Analysis of the apo bacterial structure (BpGCDH; PDB entry 3eom) overlaid with the human ternary complex with FAD and the substrate mimic *S*-4-nitrobutyryl-CoA (NBC) (hGCDH; PDB entry 1sir; Fu *et al.*, 2004 ▶) reveal structural consequences of this loss of cofactor. In all four protomers of BpGCDH, electron density was too poor to model from Thr139 to Asp145. This loop extends from the pseudo-β-barrel region into part of the FAD-binding site, as seen in Thr136–Asp143 of hGCDH (Fig. 3 ▶), acting as a wall to separate the diphosphate of FAD from the alkyl pantothenate moiety of glutaryl-CoA (Fu *et al.*, 2004 ▶; Rao *et al.*, 2007 ▶). In a different region of the human complex, a short α-helical turn comprises part of the flavin-binding site and the adjacent protomer interface. This helical character is lost in two of the four protomers of the apo BpGCDH structure (Fig. 3 ▶), further suggesting that FAD binding helps to maintain secondary structure at crucial protomer protein binding surfaces.

**Figure 4:**
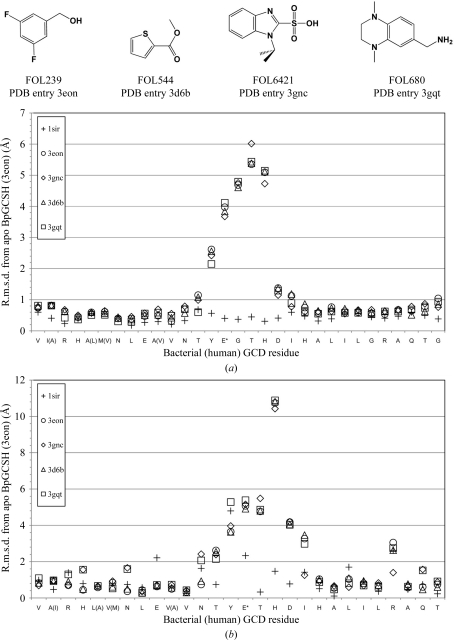
Root-mean-square deviations from the apo structure of *B. pseudomallei* GCDH for (*a*) main-chain and (*b*) side-chain atomic coordinates of human GCDH as well as fragment-bound structures of BpGCDH near the catalytic active site. (*a*) The backbone residues of the human GCDH–FAD–NBC ternary complex are structurally similar to those of apo BpGCDH. Binding small molecules causes backbone conformational changes which deviate from the apo BpGCDH structure. (*b*) The side-chain deviations between the apo bacterial structure and the human ternary complex tend to be smaller than those observed between apo and fragment-bound structures of BpGCDH. PDB codes are listed in the legend and the structures of all four GCDH-binding fragments are shown at the top. The alignment was performed using the *SSM* superposition function from the *CCP*4 program suite (Krissinel & Henrick, 2004 ▶; Winn *et al.*, 2011 ▶).

**Figure 5:**
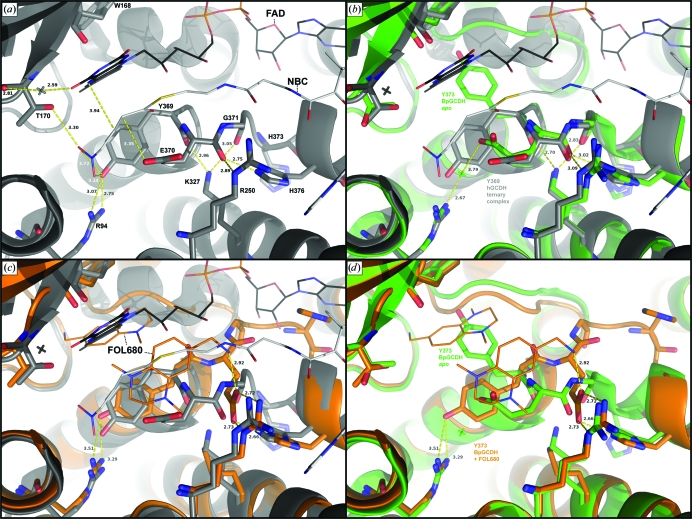
(*a*) Active site of the human ternary complex (PDB entry 1sir; Fu *et al.*, 2004 ▶), showing key interactions between FAD (black), the substrate mimic NBC (white) and key protein residues (gray). (*b*) Overlay of the active site in (*a*) with that of apo GCDH from *B. pseudomallei* (green; PDB entry 3eom), highlighting the nearly 180° rotation of Tyr373 in the bacterial structure relative to Tyr369 in human GCDH. (*c*) Overlay of the active site in (*a*) with that of the bacterial structure bound to two molecules of FOL680 (orange; PDB entry 3gqt). Backbone devations occur downstream of the catalytic glutamate, while Tyr373 is rotated back to the position seen in the human complex. (*d*) Overlay of the apo and FOL680-bound bacterial GCDH active sites.

Root-mean-square deviations (r.m.s.d.s) for main-chain atomic coordinates between the human ternary complex and the bacterial apo structure are small (<0.5 Å) near the catalytic glutamate residue (Fig. 4 ▶). Side-chain rotamers between the two enzymes also tend to be well conserved along this sequence, with the exception of Tyr373. During catalysis, this tyrosine hydrogen bonds to Arg97 (Arg94 in human) and interacts with the terminal carboxylate of the glutaryl-CoA substrate, which is enzymatically released as CO_2_ (Fu *et al.*, 2004 ▶). This tyrosine is also important for proper positioning of the substrate, creating a hydrophobic lining for the bottom of the active site (Fu *et al.*, 2004 ▶; Rao *et al.*, 2007 ▶; Thorpe & Kim, 1995 ▶; Wischgoll *et al.*, 2010 ▶). In our apo BpGCDH structure this tyrosine is flipped nearly 180° from its position in the human complex, with a distance of approximately 9.2 Å between aromatic hydroxyls (Fig. 5 ▶). In the absence of substrate and cofactor Tyr373 no longer hydrogen bonds to Arg97. Instead, the apo structure reveals Glu370 participating in a water-mediated contact to Arg97, with the catalytic glutamate carboxyl having moved 3.8 Å away from where it sits in the human enzyme. In this new location it partially occupies the substrate-binding site, but at a distance and orientation which prevent efficient hydrogen-bonding to the Arg97-bound water (Fig. 5 ▶). Thus, the tyrosine side chain of apo BpGCDH must rotate by 180° and displace a conserved water molecule in order to attain a conformation suitable for FAD binding.

### 3.2. Fragment-bound complexes of BpGCDH

With a robust reproducible apo crystal system in hand, we conducted a fragment-based screen of BpGCDH to search for novel small molecules with affinity for either the substrate- or cofactor-binding sites. Additional structures of BpGCDH resulting from this effort were obtained by soaking apo BpGCDH crystals in drops containing our Fragments of Life (FOL) ligand collection (Davies *et al.*, 2009 ▶; Begley *et al.*, 2011 ▶) prior to data collection. Approximately 10% of all BpGCDH crystals completely dissolved when added to soaking drops containing fragment mixtures and over half did not diffract after soaking. Thus, a wide variety of small molecules appear to destabilize crystals of apo BpGCDH when present in the drop. After soaking BpGCDH crystals in 181 different mixtures, 60 unique data sets were successfully collected from primary screening, representing approximately 480 tested small molecules. Follow-up soaking experiments after primary screening confirmed the identification of four unique fragment binders for BpGCDH, which are described below. Of these, one structure (PDB entry 3gnc) with FOL6421 [1-(1-­methylethyl)-1*H*-benzimidazole-2-sulfonate] bound also contains well resolved molecules of HEPES [4-(2-hydroxyethyl)-1-piperazine­ethanesulfonic acid] located in solvent-accessible cavities of the tetrahedral interface. Despite the presence of 100 m*M* HEPES in the crystallization condition and the soaking drop, neither the apo structure nor the other fragment-bound crystals of BpGCDH showed electron density for this buffer component that was sufficient for modeling. This appears to be a pH-dependent effect as FOL6421 is the only acidic fragment in the set, it is present at high concentration during the soak and no structural differences are found to account for this case.

**Figure 6:**
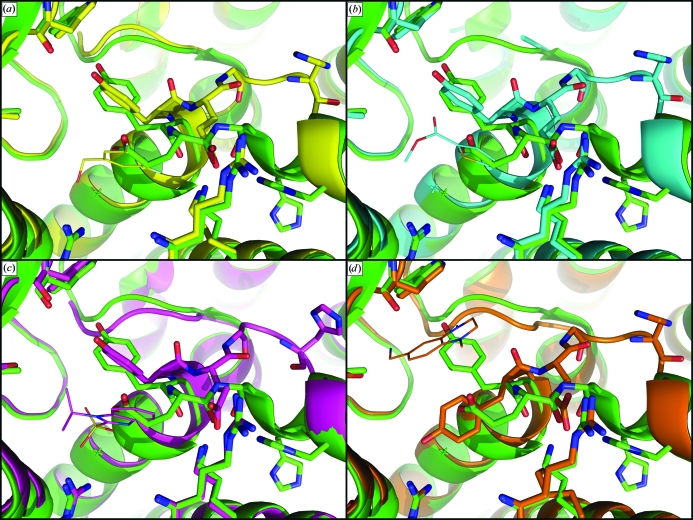
Active sites of apo BpGCDH (green; PDB entry 3eom) overlaid with complexes containing bound fragments, represented as lines. (*a*) FOL239 (yellow; PDB entry 3eon) was observed in one active site per tetramer. (*b*) FOL544 (cyan; PDB entry 3d6b) was also observed in one active site per tetramer. (*c*) FOL6421 (magenta; PDB entry 3gnc) was observed in all four active sites of tetrameric BpGCDH. (*d*) One molecule of FOL680 (orange; PDB entry 3gqt) was observed in one active site as shown, while two were observed in another active site (Fig. 5 ▶), resulting in three molecules of FOL680 per BpGCDH tetramer.

In performing its native function, GCDH binds two metabolites: the glutaryl coenzyme A substrate and the cofactor FAD. Data for 480 unique small-molecule fragments showed none that occupy the regions that bind the phosphoadenylates of either the substrate or the cofactor of BpGCDH. Although adenine, ADP and other purine analogs were contained within pools for which data were collected, electron density for these molecules was not observed after soaking and X-ray diffraction. All fragments identified by screening which bind BpGCDH (Fig. 4 ▶) occupy the pockets that bind the glutaryl and flavin moieties of the substrate and cofactor, respectively. Although much smaller than either the substrate or the cofactor, these fragments appear to stabilize crystals of BpGCDH, with a reduction in the overall *B* factor averaging 20 Å^2^ relative to the apo structure, regardless of which fragment binds (Table 2 ▶). Crystals containing FOL239 (3,5-difluoro­benzyl alcohol; PDB entry 3eon) and FOL544 (methyl thiophene-2-­carboxylate; PDB entry 3d6b) each possess one fragment bound in one active site per BpGCDH tetramer. FOL6421 (PDB entry 3gnc) was observed bound in all four active sites of tetrameric BpGCDH. Finally, a single molecule of FOL680 [(1,4-dimethyl-1,2,3,4-tetrahydroquinoxalin-6-yl)methyl­amine; PDB entry 3gqt] bound to one active site of BpGCDH (Fig. 6 ▶), while two molecules of FOL680 were observed within the active site of the tetrahedrally opposite protomer (Fig. 5 ▶), resulting in a total of three ligands bound per tetramer.

In three of the four ligand-bound complexes fragment molecules occupy the binding site for the terminal glutamate of the glutaryl-coenzyme A substrate (Figs. 5 ▶ and 6 ▶). The crystallographic water coordinated to the Arg97 side chain in the apo structure of BpGCDH remains in place when FOL544 is bound, but is displaced by FOL239 and FOL6421. In all three of these cases the Tyr373 side chain is rotated approximately 90° from its position in both the apo BpGCDH structure and the human ternary complex (Figs. 5 ▶ and 6 ▶). This new position appears to facilitate ligand binding by providing a parallel aromatic surface that aligns with the heteroaromatic rings of all three fragments. For FOL544 (PDB entry 3d6b) and FOL239 (PDB entry 3eon) this position also allows hydrogen bonding between the hydroxyl of Tyr373 and the carbonyl of Trp169, with oxygen–oxygen distances of 3.1–3.2 Å.

The Tyr373 side chain of the fourth fragment-bound complex containing FOL680 greatly differs from the other three, resulting in a position nearer to that seen in the human ternary complex (Fig. 5 ▶). This is an approximately 180° rotation from the apo state and a 90° rotation from the other fragment-bound structures. The same rotational change of Tyr373 occurs whether one or two molecules of FOL680 are present in a single active site. When one molecule is present, FOL680 occupies the binding site for the flavin ring of the FAD cofactor (PDB entry 3gqt). When two molecules are present the first occupies the same position and orientation in the flavin site, while the second binds in the substrate site closer to the pantetheine sulfur than to the carboxy-terminus of the substrate site. Like the other fragment binders, this second molecule of FOL680 is parallel to the phenyl ring of Tyr373 (Fig. 6 ▶). Insufficient density was available to capture the Tyr373 rotamer in the two remaining BpGCDH protomers of 3gqt, suggesting a low energy barrier for this side chain. Thus, the rotational mobility of Tyr373 in the absence of FAD allows the formation of at least two distinct pockets capable of binding small-molecule ligands in preformed crystals of apo BpGCDH.

Despite several structural differences between the apo bacterial structure and that of FAD-bound human GCDH, apo BpGCDH shows little backbone variation from the human ternary complex near the catalytic glutamate (Fig. 4 ▶). However, a conserved backbone deviation is observed between the structure of apo BpGCDH and those of all four fragment-bound complexes in this same region. In all four fragment-bound structures Thr376 and His377 are flipped out of the α-helix observed in the apo state, partially occupying the binding pocket for the adenosine moiety of FAD (Fig. 6 ▶). This perturbation results in a new orientation of the catalytic YEG motif. In the apo structure of BpGCDH as well as the human ternary complex, a network of hydrogen bonds occur between the backbone carbonyls of Tyr373, Glu374 and Gly375 and side-chain atoms of Arg254, Lys331 and His380 (Tyr370, Glu371, Gly372, Arg250, Lys327 and His376 in human, respectively; Fig. 5 ▶). When fragment molecules such as FOL680 (PDB entry 3gqt) bind, the main chain rotates to move all three backbone carbonyls out of position for hydrogen bonding, partially replacing them with the catalytic glutamyl carboxylate group (Figs. 5 ▶ and 6 ▶). The shift from carbonyl to side-chain carboxylate hydrogen bonding correlates with a backbone shift that constricts the FAD-binding site and places a His ring near the binding site for the adenosine of FAD (Fig. 5 ▶). Thus, the binding of small metabolite-like ligands induces a conformation in apo crystals of BpGCDH that partially fills the FAD-binding site with protein residues.

## 4. Conclusions

We have determined the first structure of an intact GCDH holo­enzyme without FAD or any small molecules present in either the substrate- or cofactor-binding pockets. Although highly homologous in structure and sequence, the apo structure of GCDH from *B. pseudomallei* revealed a loss of secondary structure and increased regions of disorder when compared with the previously determined human GCDH ternary complex. This observation, together with the relatively high number of crystals that were perturbed or destroyed during fragment soaking, suggests reduced stability of the BpGCDH crystal system, most likely owing to the absence of FAD. We have also discovered novel small-molecule GCDH binders through crystallo­graphic fragment screening and determined structures for four BpGCDH fragment-bound complexes. Structural deviations from the apo state brought about by binding small-molecule fragments reveal flexibility across certain active-site residues and appear to induce conformations which move several protein residues into the FAD-binding pocket.

Some of the mutations associated with glutaric acidemia 1 appear in the C-terminal region of human GCDH and have been shown to destabilize FAD binding *in vitro*, leading to a loss in activity (Westover *et al.*, 2003 ▶). Previous structural and enzymatic studies of GCDH have repeatedly demonstrated FAD to be a tightly bound cofactor for this enzyme. Human GCDH recombinantly expressed and purified in another laboratory by methods similar to those used here revealed FAD bound in the crystal structure, although FAD had not been explicitly added at any stage (Dwyer *et al.*, 2000 ▶; Goodman *et al.*, 1995 ▶; Fu *et al.*, 2004 ▶). Biochemical efforts to remove FAD from *Pseudomonas fluorescens* GCDH in another laboratory required multiple acid–ammonium precipitation steps, generating a very unstable protein which lost all activity if FAD was not added back within minutes (Gomes *et al.*, 1981 ▶). Despite high sequence identity to both proteins (66% to human and 85% to *P. fluorescens*), GCDH from *B. pseudomallei* can be easily separated from FAD through standard protein-purification methods, resulting in an apo protein that is stable enough for crystallization and X-ray diffraction studies. This result is consistent with weaker FAD binding to BpGCDH compared with the human or *P. fluorescens* enzymes and suggests that GCDH from *B. pseudomallei* might provide structural clues about the behavior of deficient GCDH proteins with reduced co­factor affinity.

## Supplementary Material

PDB reference: BupsA.00027.a, 3eom

PDB reference: 3eon

PDB reference: 3gnc

PDB reference: 3d6b

PDB reference: 3gqt

## Figures and Tables

**Table 1:** Data-collection statistics Values in parentheses are for the highest of 20 resolution shells.

PDB code	3eom	3eon	3gnc	3d6b	3gqt
Space group	*P*2_1_2_1_2_1_	*P*2_1_2_1_2_1_	*P*2_1_2_1_2_1_	*P*2_1_2_1_2_1_	*P*2_1_2_1_2_1_
Matthews coefficient *V*_M_ (Å^3^ Da^−1^)	2.20	2.15	2.16	2.21	2.16
Unit-cell parameters (Å)	*a* = 98.12, *b* = 106.77, *c* = 145.43	*a* = 97.41, *b* = 106.17, *c* = 144.19	*a* = 98.24, *b* = 106.10, *c* = 144.24	*a* = 98.50, *b* = 107.47, *c* = 144.31	*a* = 97.42, *b* = 106.37, *c* = 144.77
Diffraction source	APS beamline 21-ID-D	APS beamline 21-ID-D	ALS beamline 5.0.3	APS beamline 23-ID-D	ALS beamline 5.0.3
Diffraction protocol	Single wavelength	Single wavelength	Single wavelength	Single wavelength	Single wavelength
Monochromator	Kohzu double crystal	Kohzu double crystal	Asymmetric cut single crystal Si(220)	Cryocooled crystal	Asymmetric cut single crystal Si(220)
Wavelength (Å)	1.00	1.00	0.97	1.00	0.97
Detector	MAR Mosaic 300 mm CCD	MAR Mosaic 300 mm CCD	ADSC Quantum 315 CCD	MAR Mosaic 300 mm CCD	ADSC Quantum 315 CCD
Temperature (K)	100	100	100	100	100
Resolution range (Å)	50.00–2.40 (2.49–2.40)	50.00–2.40 (2.49–2.40)	50.00–2.15 (2.19–2.15)	50.00–2.20 (2.28–2.20)	50.0–1.99 (2.06–1.99)
Total unique reflections	60203	59702	81993	76333	103621
Completeness (%)	99.5 (98.2)	98.7 (98.5)	99.6 (98.5)	99.6 (97.7)	100.0 (100.0)
Multiplicity	7.0 (6.5)	7.2 (5.9)	7.0 (6.8)	7.0 (5.7)	7.2 (6.1)
Mean *I*/σ(*I*)	23.7 (2.4)	17.0 (2.2)	10.8 (2.1)	8.5 (2.3)	10.1 (2.0)
*R*_merge_†	0.084 (0.658)	0.118 (0.894)	0.072 (0.593)	0.105 (0.748)	0.073 (0.686)
Phasing method	Molecular replacement	Molecular replacement	Molecular replacement	Molecular replacement	Molecular replacement
Starting model data set	1siq	3eom	3d6b	3eom	3d6b

† *R*_merge_ = 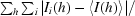 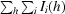.

**Table 2:** Refinement statistics Values in parentheses are for the highest of 20 resolution shells.

PDB code	3eom	3eon	3gnc	3d6b	3gqt
Resolution range (Å)	30.000–2.398 (2.460–2.398)	46.630–2.550 (2.616–2.550)	46.68–2.15 (2.21–2.15)	47.190–2.210 (2.27–2.21)	50.0–1.99 (2.04–1.99)
No. of reflections above σ cutoff in final cycle	60090	49412	81910	76240	103473
*R*_cryst_	0.213	0.191	0.209	0.212	0.202
No. of reflections for *R*_free_†	3035 (212)	2476 (179)	4105 (291)	3826 (222)	5175 (377)
Final *R*_free_†	0.264 (0.369)	0.266 (0.330)	0.255 (0.332)	0.261 (0.356)	0.238 (0.295)
Overall average *B* factor (Å^2^)	60.4	42.6	37.4	36.5	38.3
Average ligand *B* factor (Å^2^)	—	74.2	48.51	58.28	43.64
No. of protein atoms	11741	11681	11546	11436	11525
No. of ligand atoms	—	10	114	9	42
No. of solvent atoms	167	206	210	500	286
Total No. of atoms	11908	11897	11870	11945	11853

† *R*_cryst_ = 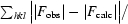 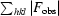. The free *R* factor was calculated using the 5% of the reflections that were omitted from the refinement).
